# Energy performance optimization of BIPV windows in dust-prone semi-arid climates

**DOI:** 10.1038/s41598-026-53426-2

**Published:** 2026-08-24

**Authors:** Isra Shorsh Muhammed, Salahaddin Yasin Baper

**Affiliations:** College of Engineering, Salahaddin University–Erbil, Erbil, Iraq

**Keywords:** Building-integrated photovoltaics (BIPV), Dust accumulation, Soiling mitigation, Secondary façade layer, Hydrophobic coating, Semi-arid climate, Energy science and technology, Engineering, Materials science

## Abstract

Dust accumulation remains a persistent constraint on the energy performance and durability of building-integrated photovoltaic (BIPV) windows in semi-arid environments. This paper assesses a facade-scale system of enhancing the operation of BIPV glazing in dusty and high-irradiance climates by introducing interventions of secondary-layers with protective, optical, thermal, and anti-soiling capabilities. The Smart Health Tower has been chosen as a case study in Sulaymaniyah, and a digital workflow was created with Rhino, Grasshopper, Ladybug, and PVsyst to evaluate the current used system and an extended full-façade baseline. As-built upper-window BIPV installation was initially used in tool-to-reality validation with the simulated annual electricity production of 321,685 kWh/year against the reported 321,000 kWh/year with is a deviation of + 0.21. After validation, the model was then extended to a complete-facade BIPV system featuring an active photovoltaic area of 5,814.6 m². The established no-layer monocrystalline baseline generated 2,478,305 kWh/year and the retained annual improvement were: optical film layer 3.0%, anti-UV/anti-scratch layer 5.8%, modular design logic 6.0%, thermal-resistant layer 8.0%, and hydrophobic coating 13.0%. The LSC based concentrating layer had the greatest projected output, 3,543,974 kWh/year, which is equal to a 43.0% increase over the baseline. These findings show that the performance of BIPV windows in dusty semi-arid environments can be significantly enhanced by means of the design of the secondary-layer at the facade, rather than photovoltaic integration alone. Simultaneously, the research identifies that the baseline case was explicitly validated, but the secondary-layer cases were modelled as literature-based comparative projections, rather completely resolved optical-thermal models. In general, the research adds a facade-based approach to the methodology of improving the energy efficiency of BIPV windows in semi-arid urban environments prone to dust.

## Introduction

BIPV windows have great potential in embedding the renewable energy generation in the building envelope, although its performance in hot dusty conditions is still limited by the environmental conditions that are not adequately investigated in the research and practice. The primary barrier in semi-arid areas is dust deposition since deposited particulates lower optical transmittance by reflecting, absorbing, and scattering solar radiation to the photovoltaic layer, thus lowering the amount of solar radiation reaching the photovoltaic layer^1^ It has been experimentally revealed that even relatively low dust density can cause a decrease in short-circuit current, maximum power, and general efficiency, and^2^proved that the transmission loss is highly dependent on the tilt angle, orientation, and airflow. In more extreme conditions such as deserts^3^, found high efficiency losses when there was a heavy deposition, which confirms the vulnerability of photovoltaic glazing to soiling in arid and semi-arid environments. This issue is increased on vertical and near-vertical surfaces by the lack of rainfall cleaning and airflow and transport patterns of particles that may lead to uneven but consistent deposition over time^4–6^. The optical performance penalty is not the only one: dust can also enhance thermal loading by changing the radiative properties of surfaces, and adding to the localized heat deposition, and a higher operating temperature can also lower electrical conversion and can enhance inward heat transfer through the envelope^7^. According to^7^, one of the key drawbacks of BIPV windows is thermal loading, which is in line with the previous research that observed that the integrated photovoltaic performance is determined by the operating temperature, the tilt angle, the level of irradiation, and the conditions of passive heat dissipation^8^. Semi-arid climates thus exert a compound stress state whereby dust deposition, intense solar radiation, high ambient temperature and scarce rainfall are all factors that reduce electrical efficiency and performance of the facade. Meanwhile, BIPV window technologies have improved significantly in terms of material innovation, optical selectivity and integration into the facade. There have been studies on transparent conductive assemblies, semi-transparent photovoltaic glazing, luminescent solar concentrator (LSC) windows, and spectrally selective energy-harvesting window systems to enhance the tradeoff between electricity generation, visible transmittance, and occupant comfort^9–11^. High-transparency window concepts, which have photovoltaic output, were developed by^10^, spectrally selective energy-harvesting windows, based on CIS photovoltaic modules and luminescent materials, were proposed by^11^, and the authors of^12^demonstrated that concentrator-integrated window BIPVT and spectral-splitting technologies Complementary review studies also shows that optical redirection, selective transmission, and spectral management can be used to improve building-integrated solar performance, although architectural integration, maintenance, cost and long-term practicality are also significant limitations^13^. However, transparency, thermal performance and optical efficiency remain the primary focus of most BIPV window research, and dust is simply indirectly addressed or considered a maintenance problem. However, dust has been named as one of the most essential reasons of the loss of photovoltaic performance^4^ demonstrated that the efficiency decrease is determined by the type of dust, concentration of the particles, climate, and exposure time, whereas^5^ focused on the role of the particle morphology, moisture, tilt angle, and exposure period. These results show that soiling cannot be considered as a secondary concern of operation in BIPV facades in dust-prone areas but as a key design parameter. The current mitigation measures may be broadly classified into cleaning-based, surface-treatment, and protective or modified surface-layer^4,5,14^. Manual cleaning is still used, yet it is labor-intensive, requires water, and is not safe and cost-effective to use on high-rise facades, particularly when dust is likely to re-accumulate quickly^15,16^. Automated or robotic cleaning can minimize manual work, yet the use of this type of cleaning on vertical glazed envelopes is limited to the conditions of access, articulation of the facade, and the complexity of its maintenance^14,15^. Glazing is better compatible with surface-based solutions: hydrophobic and anti-soiling coatings have been investigated to decrease the adhesion of dust and curb the performance degradation^17–21^. Anti-UV and anti-scratch coating can also serve to maintain the stability of materials and the optical quality retained, but the literature more frequently provides the results of durability than the annual energy savings of such coating^22–24^. Table 1 provides a summary of these methods based on mechanism, benefits, constraints and applicability to high-rise BIPV windows. These strategies are however fragmented as far as a facade-engineering is concerned. The cleaning solutions are operation-based and rely on access logistics, water, safety processes, and frequent maintenance expenses, and the coating-based solutions are susceptible to optical aging, abrasion, ultraviolet degradation, and unpredictable long-term performance under harsh conditions^18,22,23^. In this way, although both techniques have some degree of advantage, none of them will be able to meet the combined architectural, environmental, and operational requirements of high-rise BIPV windows in semi-arid environments. Mitigation of dust must not be, however, dealt with as a cleaning issue, but as a design issue of facades, relating to the geometry, layering, and optical behavior of the envelope assembly itself. This is a facade-based standpoint that is supported by studies on BIPV facade design and high-tech glazing system^25^. Demonstrated that the changes in the geometry of the glass, the distance between the cells, the coverings, and the composition of the system could enhance the performance and minimize the obstacles to the adoption of BIPV. Similar studies on optimization of facades and integrated solar-envelopes also highlight the significance of glazing, shading, and photovoltaic technology coordination to enhance the energy performance of buildings at the building scale^26–28^. Within this context, the concepts of secondary-layer are particularly applicable. A protective or optically functional layer placed over the front of photovoltaic glazing has the potential to reduce direct exposure of dust onto the active surface, alter the thermal interaction of the facade, and enhance the use of the solar utilization when it is diffusely or obliquely irradiated. Studies in the field of concentrators, optical films, and luminescent systems have already shown that additional layers on the facade can redirect, filter, or spectrally control incident radiation to enhance the use of photovoltaic^11,12,29,30^. This is particularly relevant where dusty climates in which the suspended and deposited particles will increase optical scattering and change the direct-to-diffuse irradiance ratio at the glazing surface. Nevertheless, although this concept is overlapping, the literature still lacks the studies that can compare a secondary layer integrated into the facade before BIPV glazing as a hybrid approach to dust-reduction and performance-enhancement of semi-arid environments. Based on this, the paper explores the effectiveness of a secondary-layer of strategy in front of BIPV glazing in semi-arid and dust prone regions. It assesses the potential of a secondary protective or optically functional layer to enhance the performance of BIPV windows through exposure to dust, better use of solar energy in less ideal irradiance conditions, and more stable long-term operation using a façade-scale case study. The research thus covers three objectives that are interrelated and that are to minimize the exposure of the main photovoltaic glazing to dust deposition, enhance the optical use of incident and diffuse solar energy, and supporting more stable long-term façade operation. The study provides an architecturally integrated foundation of enhancing the BIPV window design in extreme environmental conditions by connecting the behavior of dust, facade-layer logic and BIPV window performance into a single simulation platform.

**Table 1:** Comparison of commonly used dust mitigation strategies for BIPV/PV systems.

Strategy	Mechanism	Advantages	Limitations	Key constraint in high-rise semi-arid application	Applicability to high-rise BIPV windows	Relevance to current study
Manual cleaning	Periodic washing or wiping of exposed PV/glazing surfaces	Simple and widely understood; low technological complexity	Labor-intensive; water-dependent; irregular cleaning intervals	High labor demand and access difficulty on tall façades	Limited	Represents the conventional maintenance approach but not an integrated façade solution
Robotic/automated cleaning	Mechanized brushing, wiping, or water-assisted cleaning using mobile devices	Reduces manual labor; can improve cleaning frequency	Higher capital cost; integration difficulty; maintenance of cleaning device	Façade access, articulation, and vertical-envelope compatibility	Moderate to low	Relevant as an operational strategy, but not as a passive envelope-integrated measure
Electrodynamic/electrostatic cleaning	Electric field used to remove or displace dust particles from the surface	Water-free; non-mechanical; promising for dusty environments	Technological immaturity in architectural use; integration and durability concerns	Scalability and transparent glazing integration	Moderate	Relevant conceptually, but currently limited for large-scale façade deployment
Hydrophobic/anti-soiling coatings	Reduced surface energy minimizes dust adhesion and facilitates self-cleaning	Passive approach; visually compatible with glazing; no moving parts	Coating degradation; abrasion; UV aging; variable long-term effectiveness	Durability and uncertain long-term optical performance	High	Directly relevant as one of the protective strategies assessed in this study
Protective secondary glazing/film layer	Outer layer shields the active PV glazing from direct dust exposure	Can combine physical protection, optical control, and thermal moderation	Added cost; installation complexity; cavity maintenance may be required	Interlayer cleaning and added façade complexity	High	Central to the current study
Optical concentration/redirection layer	Redirects or concentrates incident radiation toward active photovoltaic regions	May improve solar utilization under diffuse or oblique incidence	Optical losses; angular sensitivity; thermal penalties; higher design complexity	Need to balance gain, heat, and optical realism	Moderate to high	Central to the current study

## Literature review

### BIPV systems: definitions, typologies, and façade integration

**Fig. 1:**
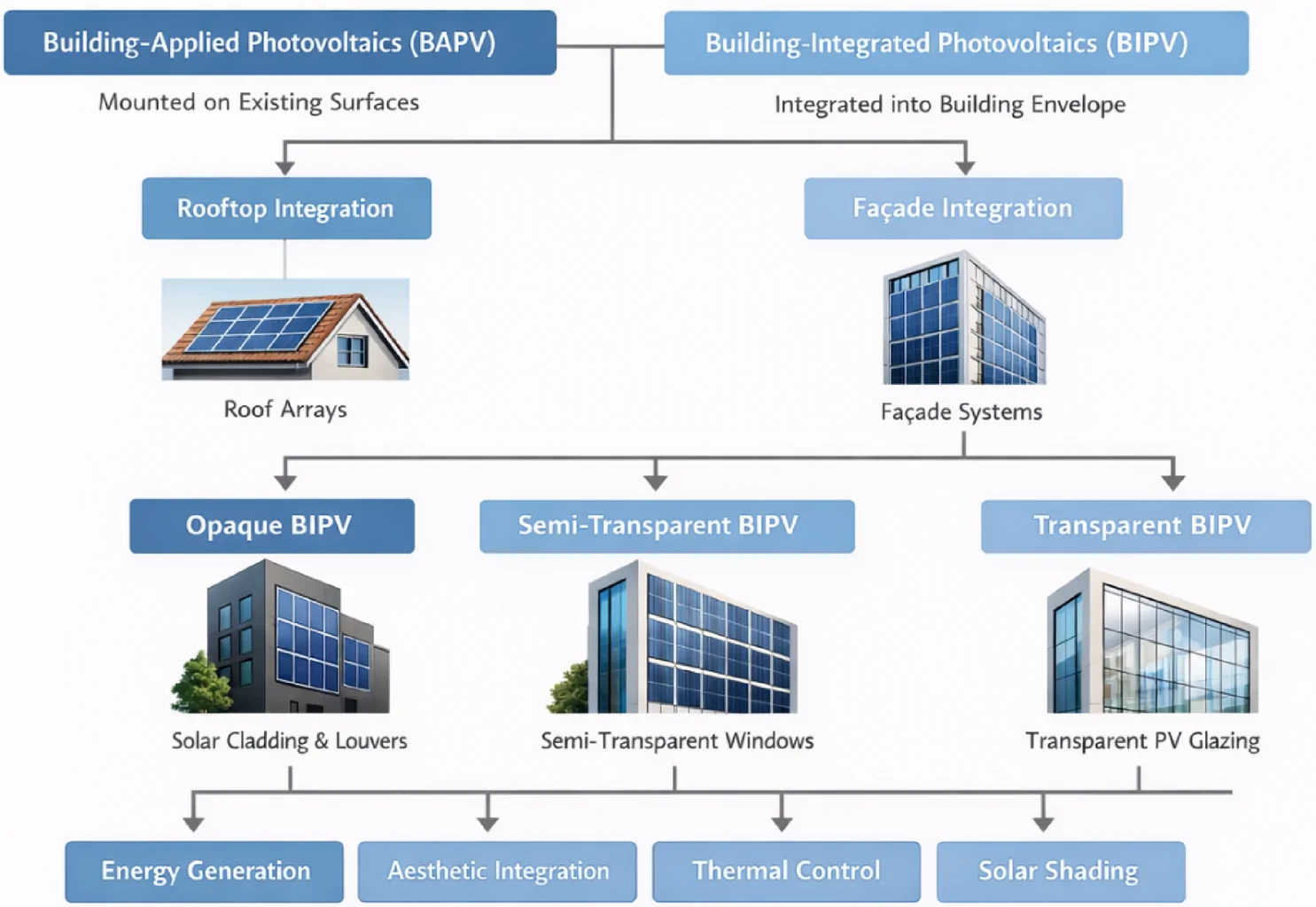
Categorization of building-integrated photovoltaic (BIPV) systems based on type of integration and transparency of the building envelope. Source: Authors’ own conceptual synthesis based on relevant BIPV literature.

**Fig. 2:**
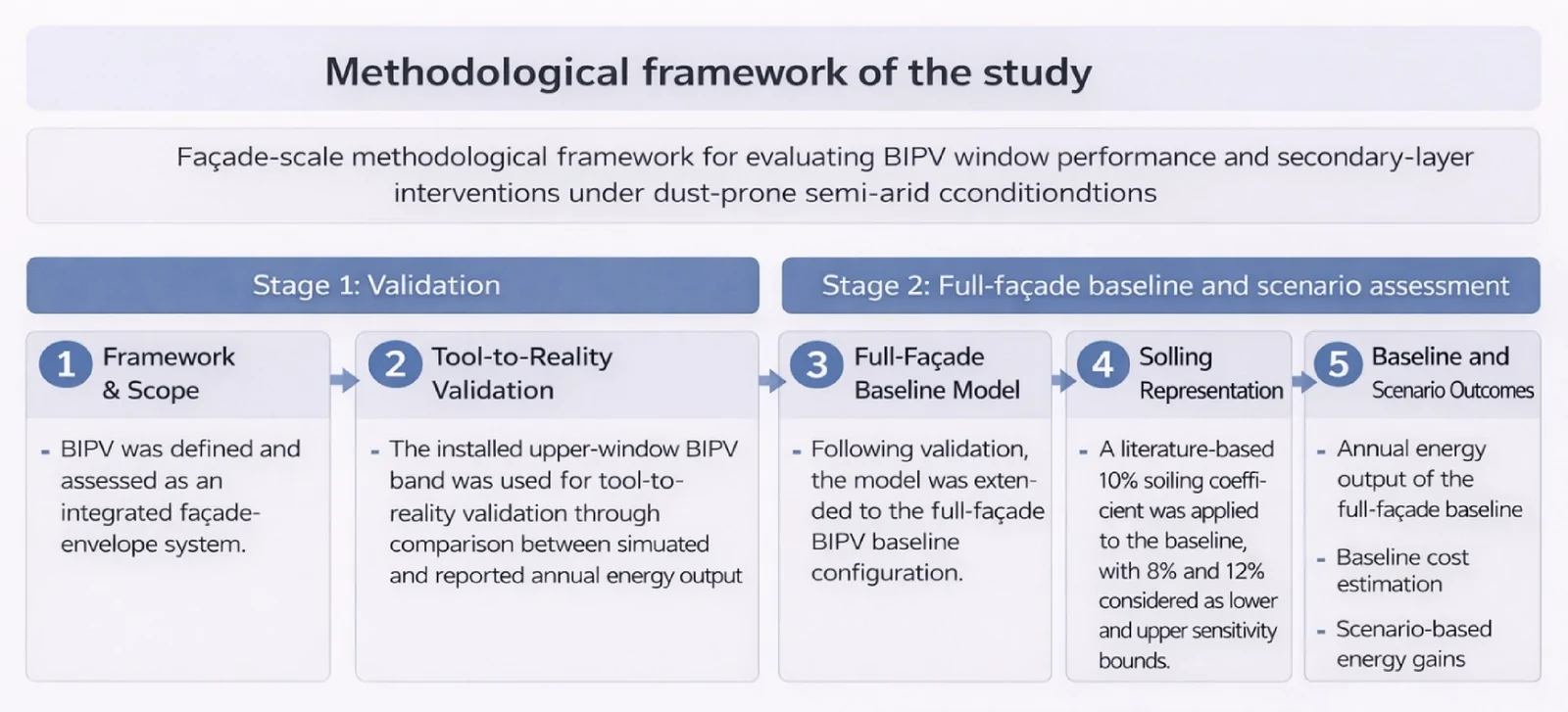
Methodological framework of the study.

**Fig. 3:**
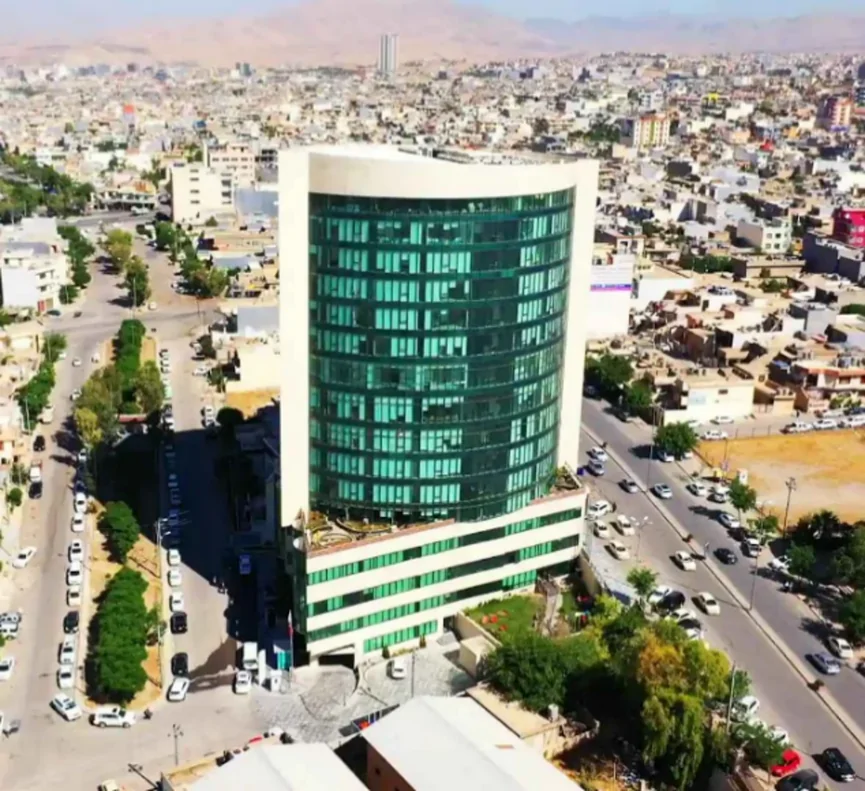
Smart Health Tower in Sulaymaniyah, showing its curved glazed façade. Source: Smart Health Group official website.

**Fig. 4:**
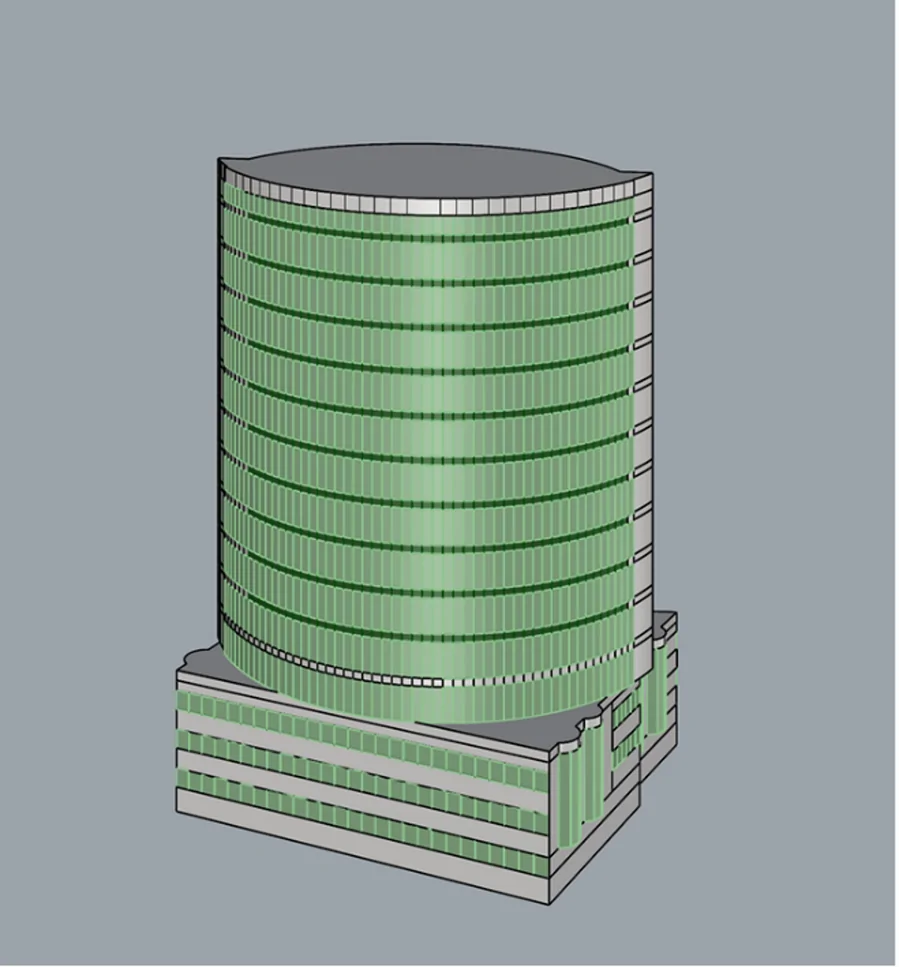
Recreated 3D model of the Smart Health Tower showing the façade configuration used in this study. The dark green horizontal belt or band illustrates the building’s actual transparent BIPV application, while the light green glazing surfaces show the simulated extension to a full-façade BIPV envelope.

**Fig. 5:**
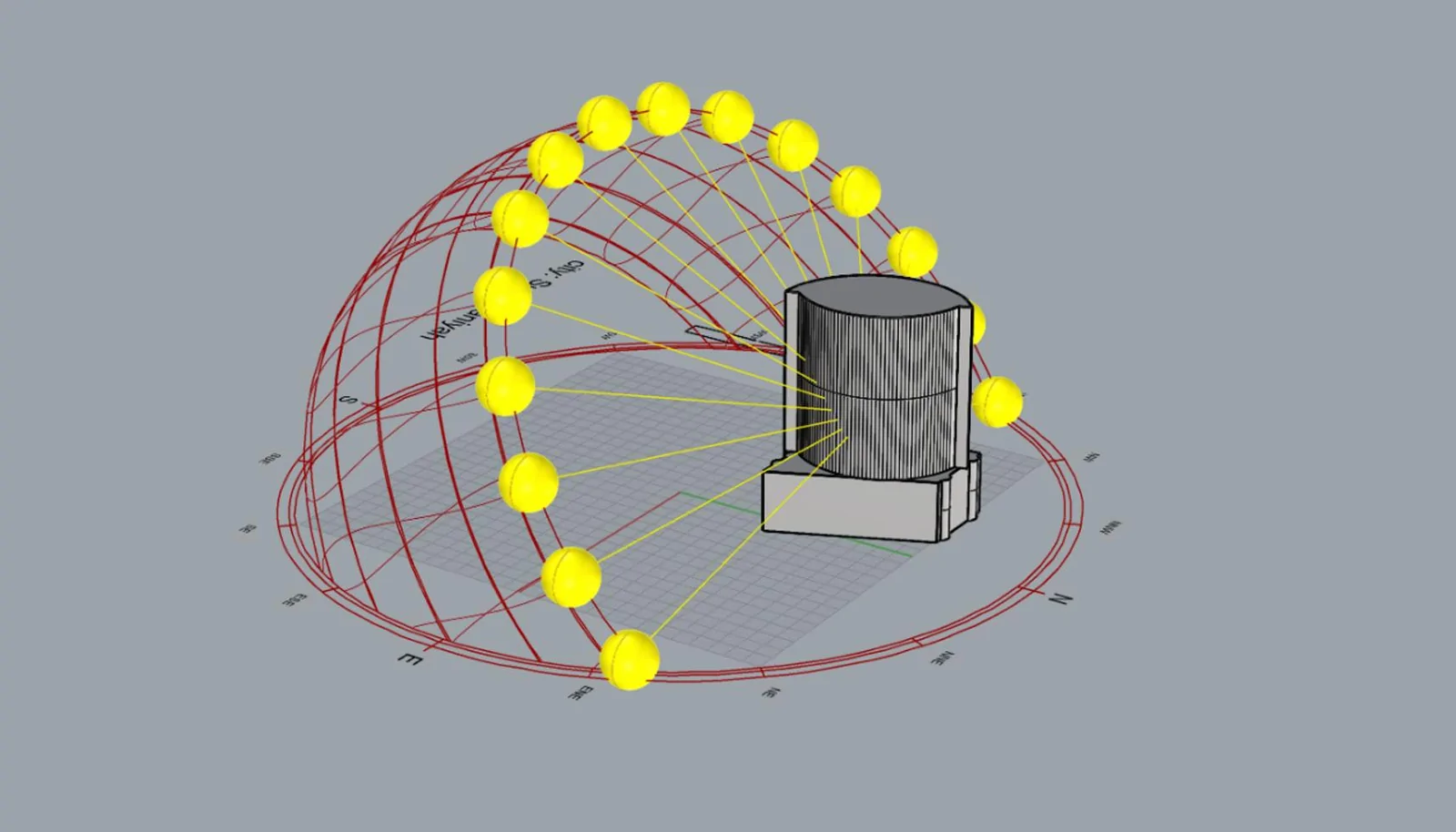
Three-dimensional solar-path visualization showing seasonal sun positions around the Smart Health Tower. Yellow spheres represent selected hourly sun positions, while the connecting vectors indicate the direct solar ray’s incident on the curved façade. This model demonstrates the annual solar exposure pattern used to drive façade-level irradiance analysis.

**Fig. 6:**
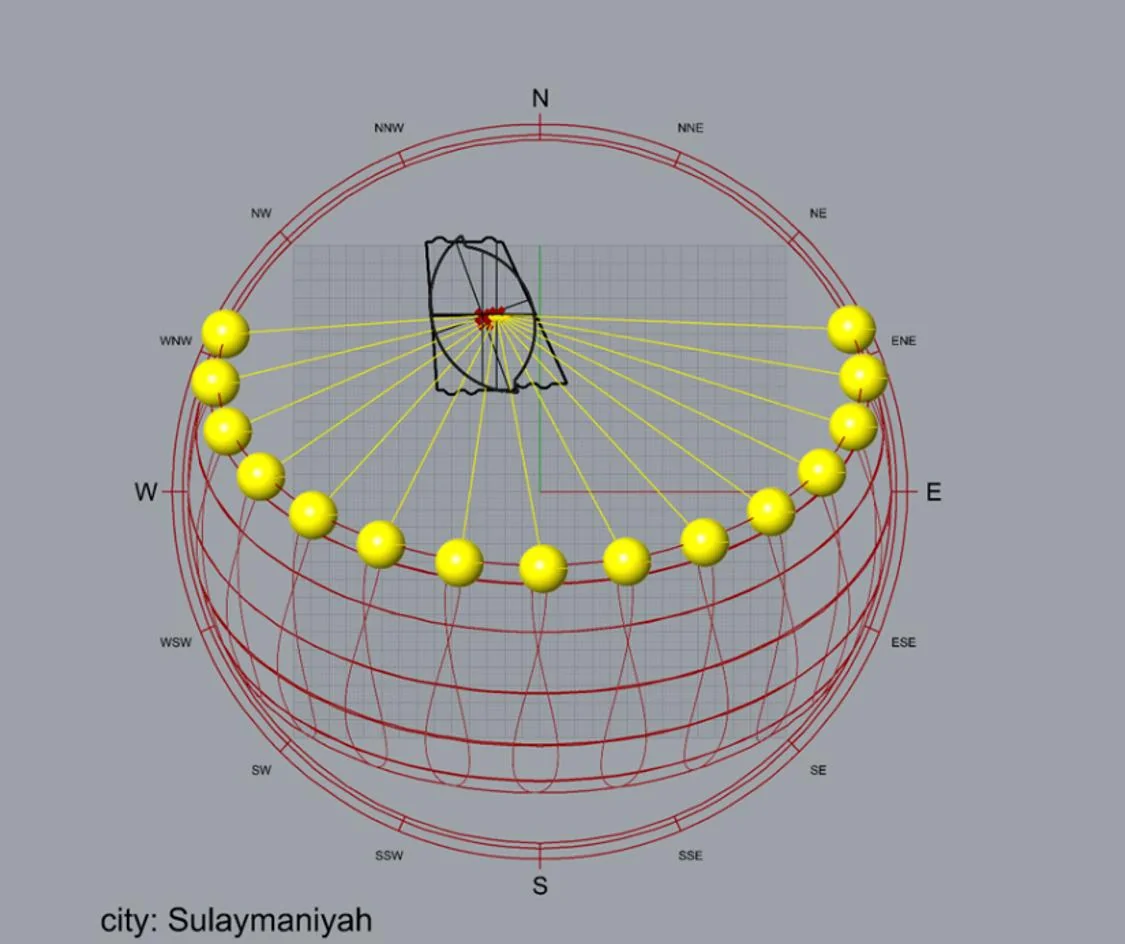
Top-view solar-path diagram for the case study, showing the azimuthal distribution of annual sun positions. The yellow sun markers and radial vectors are used to show the intersection points of the direct beam radiation on the tower footprint which can be used as inputs of orientation-specific irradiance to the simulation workflow.

Building-integrated photovoltaics (BIPV) are photovoltaic systems that are built into the building envelope, and which have both construction and energy-generation functions. BIPV can substitute traditional envelope materials and can be built into walls, roofs, glazing systems, shading systems, and other building elements unlike building-applied photovoltaics (BAPV), which is mounted on existing surfaces^31,32^. In this respect, BIPV is not judged by the electricity generation, but also by the contribution to the envelope performance, architectural integration, and environmental response^27^; IEA PVPS Task 15, 2025). The most common groups of BIPV systems include those based on location and those based on optical or functional purpose. They are separated by application into roof-integrated and façade-integrated. BIPV that is built into the roof typically has a better solar exposure and tilt, whereas BIPV built into the facade, despite less incidence angles, has a larger available surface area in high-rise and dense urban buildings where the available roof area is smaller than the demand^33^. BIPV can be either opaque, semi-transparent or transparent by functionality. Panels and spandrels with cladding are generally clad with opaque systems, whereas windows, skylights, atria, and glazed curtain walls are usually clad with semi-transparent and transparent systems where the production of electricity should be balanced with the daylight transmission, visual connection, solar control, appearance of the facade, and comfort of the occupants^34,35^. The increased interest to BIPV is predetermined by the need to have low-carbon and energy-efficient buildings. Since the photovoltaic elements are integrated into the envelope, BIPV integrates the generation of renewable energy with material replacement and integration into the architecture, which is also known as one of its main benefits^27^; IEA PVPS Task 15, 2025). This is particularly important in facade application this multifunctionality is especially significant as the envelope has to meet the environmental, indoor-comfort, and architectural demands at the same time. BIPV integrated into the façade, however it cannot be viewed as an extension of traditional photovoltaics to the vertical plane. Once it is incorporated into the facade, it is conditioned by geometry, orientation, thermal relationships with the envelope, and general architectural reason^25^, reveal the enhancements of the architectural adaptability due to the differences in geometry, spacing, and system composition, whereas Cuce and Cuce^8^ reveal that the electrical performance is not only dependent on the irradiation, but also on the operating temperature, the tilt angle, and the passive heat dissipation. This is especially significant in façade-parallel applications, where envelope integration can increase thermal accumulation and decrease production if the heat is not suitably managed. Based on this, BIPV facade systems should be conceptualized as photovoltaic technologies and building-envelope systems whose functionality relies on the interaction of architectural design, climatic context, thermal performance, and functionality. This is needed prior to analyzing the individual benefits, drawbacks and performance issues of BIPV windows in semi-arid regions with dust. Figures 1, 2, 3, 4, 5 and 6.

### Architectural advantages and architectural performance limitations of facade-integrated BIPV

Building-integrated photovoltaics (BIPV) are used to generate electricity and perform the envelope role, thus not being applied to existing surfaces, but instead replacing the standard material of facades or roofs^31,32^. This provides BIPV with a greater architectural and environmental impact than stand-alone PV in terms of on-site generation of renewable energy, material replacement, and envelope multifunctionality^27^; IEA PVPS Task 15, 2025). It is also particularly valuable in dense urban and high-rise settings since in many cases, rooftop photovoltaics cannot contribute as much due to the limited availability of a rooftop space, but the facade can offer a greater active area to the sun exposure^33^. BIPV is also flexible in terms of architecture, as the photovoltaic elements can be incorporated into the composition of the facade with a variety of transparency, geometry, distance, and surface rhythm instead of being perceived as independent technical elements^25^. BIPV can also be used in glazing to help regulate the environment (BIPV can act as a shading surface or solar filter, or a semi-transparent envelope layer that can balance the amount of daylight entering and the amount of electricity produced)^7,34^. Simultaneously, the BIPV embedded in facades has significant limitations, which make it different to rooftop PV. Vertical and near-vertical facades tend to work with less solar incidence, and thus performance is highly determined by the direction, the geometry of the facade, and climate^33^. Another significant drawback is thermal behavior since close envelope integration may limit heat dissipation; the operating temperature, tilt, and passive cooling conditions are thus another factor in BIPV performance^7,8^. Moreover, systems embedded in facades are very sensitive to the effects of shading, urban obstruction, and transparency, daylight, and optical quality which may decrease photovoltaic space or modify optical performance^25,34^. The trade-offs are especially high in semi-transparent and transparent BIPV, in which the production of electricity has to be compromised with visible-light transmission, solar control, and occupant comfort^7,34^. The cost, complexity of installation, maintenance, and long-term durability further restrict the wider adoption of the photovoltaic layer as the photovoltaic layer is required to be synchronized with waterproofing, structural integration, replacement strategy, and service access as a part of the building assembly^13,27^. In general, the concept of façade-integrated BIPV can be interpreted not as a renewable-energy system, but as an envelope technology of a building that can be considered in terms of its advantages and disadvantages as the result of the combination of energy performance, thermal performance, facade design and durability.

### Semi-transparent and transparent BIPV window technology

Semi-transparent and transparent BIPV windows are photovoltaic glazing systems that are able to transmit daylight, solar control and produce electricity on-site within a single façade element. In contrast to opaque PV modules, they work based on a multi-objective balance where electrical production has to be balanced with the visible light-transmittance, façade appearance, the visual comfort inside the building, and the control of solar gain^7,34^. The semi-transparent BIPV is generally developed by using controlled cell spacing, patterned deposition, or partial absorber coverage where some part of the visible spectrum could be permitted to pass through maintaining photovoltaic operation^34^. Transparent BIPV builds on this principle by spectral selectivity and optical routing, such as luminescent solar concentrator (LSC) principles and spectrally selective surfaces that separate non-visual radiation to photovoltaic areas while maintaining visible transparency^10,11,29^. These systems are also gradually being seen as multifunctional systems of facades, which can combine solar conversion, daylight control, and lower solar heat gain with the help of improved glazing assemblies, transparent conductive materials, and spectral filtering strategies^9,34,36^. They are also, however, still limited by the basic transparency efficiency trade-off, as increased visible transmittance tends to decrease photovoltaic performance. That is why^37^, claim that transparent PV glazing should be considered within the electric, thermal, and daylighting domains instead of assessing it based on the energy generation. On the facade aspect, these technologies are valuable as they add photovoltaic functionality into the glazed visual-sensitive areas without complete occupying the daylight or the exterior view. Nevertheless, their sensitivity to dust and abrasion, optical aging, and non-uniform fouling also makes them particularly vulnerable to such factors, as these may result in poor visual and photovoltaic performance^7,34,37^. This makes them of specially relevance to the study of dust-related loss of performance and secondary-layer strategies in semi-arid climates.

### Soiling and climatic stressors in semi-arid dusty areas

The semi-arid areas present a difficult environment to BIPV windows since high levels of solar irradiation is combined with prolonged dryness, which is coupled with low rainfall and dusty environments. This generates good solar potential but accumulated soiling, especially on the glazing of the facades and minimal natural cleaning, which results in the simultaneous action of dust deposition, thermal loading, and high-intensity solar exposure on the photovoltaic envelope^4,5^. Regional and local sources, such as soil erosion, traffic, construction activity, and long-range transport of particulate matter determine dust behavior, which results in the formation of particles of various sizes, compositions, and optical effects^2,5,6^. In turn, the severity of soiling is determined by the properties of particles, roughness of the surface, moisture, wind regime, and duration of the exposure and thus differs depending on the location and facade exposure and climate^4,5^. Particularly tilt angle and orientation^2^, demonstrated that the dust has a considerable influence on the solar-glass transmittance and that the effect is dependent on the tilt, airflow, and exposure. Vertical surfaces can be not as susceptible to dust as low-slope surfaces; however, in dusty (low-rainfall) conditions, they are also vulnerable^4,6^. The adhesion of fine particles can be due to electrostatic forces, surface-energy forces, bonding by moisture, and surface irregularities, and the wind can be depositing and removing dust based on the aerodynamics of the facade and urban context^2,5,6^. This can cause an uneven deposition on the envelope in high-rise buildings. Thermal stress also interacts with dust, whereby it decreases optical transmission and changes radiative aspects of surfaces, which may enhance efficiency losses related to temperature when environments are hot and highly irradiated^7^. In general, soiling in semi-arid areas is an optical, thermal and facade-performance issue which is influenced by dryness, particle composition, wind, orientation, tilt angle and surface properties.

### Effects of dust on the PV and BIPV window performance

Photovoltaic performance is degraded by dust deposition by both optical, electrical, and thermal mechanisms, and is particularly critical in glazing-based BIPV since surface transparency, solar transmittance and optical quality are directly affected. Scattered and reflected incident radiation reduces the energy reaching the active layer and decreases output due to the deposited particles absorbing it^1,4^. The effects of BIPV windows go beyond the electricity production to the transmission of visible light, daylight, visual quality, and solar-control performance of the facade^7,34^. The experimental evidences demonstrates that significant losses can be caused even at the lowest deposition levels^4^:, have found that a dust load of 4 g/m² can decrease electrical efficiency by up to 40%, thin layers of urban pollution have been observed to decrease it by up to 20%, and in some instances even 40%. This electrical response is not necessarily linear, as dust can have different effects on current, voltage, fill factor, and operating stability, but short-circuit current is frequently the most sensitive, and voltage may react less at moderate deposition^4,5^. At heavier and/or non-uniform fouling, though, the maximum power and the overall efficiency drops at a steeper rate, which becomes especially relevant when considering the case of façade-integrated BIPV glazing where wind-induced deposition, curvature of the facade and localized exposure can cause uneven patterns of soiling. Reported losses also vary widely with climate, rainfall, exposure period and tilt angle. According to^4^, average daily energy loss can be up to 4.4% and dry seasons can increase daily losses to 20% and efficiency decline in hot rainless climates can be as high as 0.2% per day the same as the productivity losses of 56.2% annually. In the literature reviewe, the daily losses of PV power are between 0.05% and 1.15% and monthly losses of efficiency may be more than 80% in extreme cases. The results per country are 17.4% monthly efficiency reduction in Egypt, 19.8% in Saudi Arabia, 13% in Mexico, 17% in Kuwait, 7% in another Saudi case, 60.6% in a severe Iranian case, 31–35% in productivity loss in Jordan, 0.4–0.8% in daily loss in Qatar which corresponds to 12–24% in monthly loss without cleaning, and 5.86% annual energy deterioration on Crete-greece^4^.

Tilt angle is one of the aspects that are still significant^4^, refer to Egyptian experiments that found that deposited dust reduced to 15.84 g/m² at 0 o of tilt and 4.48 g/m² at 90 o of tilt, with a loss of permeability between 52.54% and 12.38%; they also note that the voltage can be reduced by over 6% Though near vertical BIPV facades can hold less dust than low slope ones, they are susceptible in dusty urban conditions where there is little rainfall and cleaning is not done too often. A thermal penalty is also caused by dust, which alters surface radiative behavior and raises local surface temperature, which further decreases efficiency^4^^[,7^. Dust under hot semi-arid conditions must be understood, in other words, that it is a coupled optical-electrical-thermal degradation process and that it is a key determinant of the operational and architectural performance of BIPV windows^7,8^.

### Current dust mitigation measures of PV and BIPV glazing

Dust-reduction strategies of photovoltaic systems can be broadly classified into cleaning, surface-treatment and protective-layer methods, although their applicability to façade-integrated BIPV glazing is less favorable than to other systems, where transparency, access, durability, and long-term optical performance are all critical^4,5^. The most established cleaning technique is manual cleaning, but it is not as effective because of the labor requirement, use of water, re-soiling, and ineffectiveness on high-rise facades^15,16^. Regularity can be enhanced with automated and robotic cleaning, although they cannot be applied to the architecturally complex glazed facades and create the additional cost and maintenance burden^4,14^.

Surface-treatment methods are more compatible with glazing. Anti-soiling, anti-reflective, textured, hydrophobic, and self-cleaning surfaces are designed to minimize the adhesion or loss of dust or transmission loss of optical quality. The anti-soiling coatings in desert environments were found to improve performance^18^, the textured and anti-reflective glass surfaces were identified to be promising but highly conditional on climate, durability, and exposure^17,38^. The anti-UV and anti-scratch treatments are also applicable since the retained transmittance and long-term stability are vital in exposed glazing systems^22–24^.

Strategies to enhance the retained performance are operation-and-maintenance strategies, which enhance cleaning schedules, inspection, monitoring, and service planning, and modular façade strategies which could enhance access and replacement control^15,16,35,39^. One more direction that is more facade-oriented is the application of protective or optically functional secondary layers that are placed in front of the active glazing and that can help to minimize direct dust exposure as well as have an effect of optical and thermal performance. Overall, cleaning, coatings, and O&M methods only partially solve the issue of soiling, which explains the need to pay more attention to the secondary-layer methods of facades-integrated BIPV windows to dusty semi-arid environments.

### Thermal-responsive and protective secondary-layer materials

Thermal responsive and protective secondary-layer materials are becoming more applicable to BIPV windows since they help overcome two long-standing constraints of the facade, namely thermal accumulation and surface degradation. High irradiance and ambient temperature increase the operating temperature of photovoltaic and decrease the efficiency in hot climates, and outer glazing should also be able to withstand ultraviolet exposure, abrasion, dust impact, and weathering (Liu and Wu, 2022;^7^. Research on adaptive glazing including thermotropic and electrochromic systems demonstrates that additional functional layers can be used to control solar transmission and minimize overheating and still maintain daylight functionality, which applies to BIPV since operating temperature has a direct impact on electrical yield and envelope characteristics^8^. This rationale is also backed by photovoltaic thermal-management studies Hamdan^40^ found better performance with passive cooling, and^41^, considered finned phase-change-material systems to control thermal variation in time. Façade studies also support the added outer layers. The retrofit double glazing was also found to be a significant envelope intervention by^42–44^,demonstrated that the conditions of cavities in the double-skin facades may change the air temperature, surface temperature, airflow, and overall thermal performance^45,46^, also proved that the performance of multi-layer facades is also determined by airflow, heat exchange, and the structure of the layers, rather than the properties of the PV materials in photovoltaic applications. Durability is also ensured by protective layers^22–24^, demonstrate that retained transmittance, UV resistance, and abrasion durability are the key factors in the long-term operation in extreme arid conditions. Overall, secondary layers could be seen as the aspects of the facade performance, which have the potential to moderately control heat, retain optical quality, and enhance the operational stability of semi-arid BIPV glazing.

### Proposed secondary-layer BIPV facade air cavity design

Existing literature shows that ventilated double-layer design is more appropriate in BIPV windows as compared to sealed cavity^47^, reasoned that ventilated double BIPV windows have the highest potential of energy-saving and climatic flexibility whereas^48^, found operation by natural ventilation to be the most suitable in hot climates in BIPV double-skin facade that includes an outer semi-transparent PV layer, an inner glass layer, and an intermediate cavity. Accordingly, the current study uses a compact front ventilated air cavity between the secondary protective outer layer and the BIPV glazing/window rather than a deep occupiable double-skin façade^49^, presented dimensional guidance and established that the greatest change in PV temperature was observed when the spacing was increased between 0.05 m and 0.15 m, and suggested an engineering range of about 0.10–0.15 m. according to that, the present study will use a cavity depth of 100–150 mm with 120 mm as the nominal value to balance the ventilation performance, the compactness of the facade and constructability. Architecturally, based upon detailing of the cavity, it is suggested that the cavity be proposed as a laterally enclosed vertical air gap with screened bottom and top openings, rather than a fully sealed or fully exposed open-joint structure. An open cavity would enhance the direct ingress of dust, but a complete seal would undermine the passive ventilation and add technical complexity^50^, substantiate this principle with the help of a screen-covered top-and-bottom ventilated cavity, and^51^, also confirm the appropriateness of solar double-skin facades in warm weather. Based on this, the proposed system will be a naturally ventilated, laterally enclosed front cavity with a screened top and bottom opening and nominal depth of 120 mm within a defensible range of 100–150 mm.

### Customization of Façade, multi-layered systems and performance assessment

Photovoltaics integrated into facades are subject to architectural, environmental and technical limitations that make the customization of photovoltaics to be a defining performance determinant and not a design concern. In contrast to stand-alone PV, BIPV facades have to react to energy production, daylighting, envelope structure, visual expression and construction rationality. According to^25^, geometry, transparency, spacing, and system composition are thus considered to be the variables of enhancing the architectural flexibility and lowering the conflict between energy and facade design goals. These variables affect solar exposure, self-shading, day light penetration and the general envelope character especially in high rises and urban environments^25,33^. The optimization of BIPV should thus be considered at the scale of facade-assembling instead of being considered at the material level. This reasoning is more significant in multi-layer systems. Research on the development of glazing demonstrates that outer layers, cavities, interlayers, and inner glazing together can control the solar transmission, heat gain, optical quality, and environmental resilience^52^. Therefore, a secondary layer in front of BIPV glazing, whether protective, thermal-responsive, or optically functional, should be perceived as a component of a larger strategy of facade-engineering and not as an independent addition. It is backed by the fact that facade studies have shown that retrofit double glazing can serve as a substantial envelope intervention and cavity conditions in the double-skin facades have a significant impact on airflow, temperature, and thermal behavior^42–46^, also demonstrate that electrical and thermal performance in photovoltaic multi-layer facades is also dependent on airflow, heat exchange, cavity conditions, and the arrangement of the layers. The most recent developments on modular and prefabricated facades further underline the fact that performance is connected with constructability, maintenance accessibility, replacement strategy, and long-term operation^35,39^. That is why the BIPV window systems must be considered not only in terms of electricity production, but also in terms of electrical, optical, thermal, environmental, and architectural aspects, such as daylight, solar control, usability, and compatibility with design^34,^^37^. In general, the literature upholds a multi-criteria, multi-layer BIPV systems assessment framework based on the facade scale^25,26,28,35,39,42–44,53,54^.

### Study gap and rational of the present research

The literature review indicates that the field of BIPV has advanced in the definition of the technology, integration of the facade, transparent photovoltaic glass, dust-related loss-mechanisms, and mitigation strategies^4,25,27,34^. Similar facade investigations also prove that secondary transparent materials, ventilated cavities and photovoltaic multi-layers assemblies can have a dramatic impact on thermal and electrical performance at the architecture scale^42,45^. Nevertheless, they do not provide enough integration that is required for the specific case of BIPV windows in dusty semi-arid environment. The primary gap is not the lack of the available knowledge, but the inability of the integration between the existing research directions. The existing literature on dust mitigation primarily focuses on the conventional PV, cleaning, or coating instead of the facade-integrated BIPV glazing^4^. In comparison, semi-transparent and transparent BIPV are studied in terms of transparency, daylighting, and thermal behavior, whereas dust is not considered a key performance variable of the facade^7,34^. Similarly, the significance of added layers and cavity behavior is established by facade-layer studies, but fail to directly test a front secondary layer in front of BIPV glazing as a combined strategy of dust-protective, thermal, optical and facade-performance in semi-arid environments.

## Methodology

### Methodological framework of the study

The methodology of the study was a facade-scale approach to assess the performance of BIPV windows and compare the interventions of secondary layers in the conditions of dust-prone semi-arid climate. The workflow considered BIPV as a system of envelope and combined reconstruction of facade, irradiance analysis, representation of soiling and energy analysis. This procedure was done in two phases. To validate the tools to reality, first, the upper-window BIPV band installed on the Smart Health Tower was utilized. The modelling and solar analysis of the building facades were done with Rhino, Grasshopper and Ladybug and the annual output of the simulation was compared to the reported building output. The model indicated a prediction of 321,685 kWh/year compared to the actual 321,000 kWh/year with a deviation of + 0.21. Second, the validated model was expanded to a full-baseline facade and cross-validated in PVsyst. The total active photovoltaic area of the entire facade was 5,814.6 m², which represented by 2,296 facade analysis segments. The baseline was set to 10% soiling coefficient based on literature with 8% and 12% being the lower and upper sensitivity limits. The scenarios of the secondary-layer were evaluated on a comparative basis, energy-gain-factor, where the literature-based improvement values were used to evaluate the validated baseline. The conceptual basis of the secondary-layer assembly was a ventilated double-layer facade, although the airflow in cavities, convectional heat transfer, and internal dust build-up were not explicitly modeled. Economic assessment was limited to the baseline full-façade case.

### Case study building and façade characteristics

The Smart Health Tower in Sulaymaniyah was chosen as a single case study due to the fact that it is a completely glazed high-rise medical facility in a semi-arid climate with a high amount of solar radiation, low rainfall, and frequent airborne dust. These circumstances make it a relevance case in terms of investigating the architectural and energy-performance implication of BIPV window integration in the conditions of severe environmental exposure. The building also has a real facade-integrated BIPV system comprising of 900 semi-transparent monocrystalline panels that are spread over the two curved facades (450 panels on each side) of the building with a total installed capacity of 232 kWp DC and two 110 kW Huawei SUN2000-110KTL-M0 inverter, the implemented system takes about 752.9 m² area in the upper window bands and was used as the physical reference of glazing type, transparency and logic of facade integration. One important architectural aspect of the tower is the curved vertical envelope that provides the tower with uneven exposure to the sun on the facade and necessitates the use of orientation-sensitive analysis. In that regard, the case study is a suitable architectural paradigm to evaluate the transparent BIPV windows in a high-rise glazed structure that the geometry of the facade, the environmental exposure, and the envelope interface directly interact.

### Data input and model inputs

Data collection combined architectural, climatic, and photovoltaic data to create the applied-system and full-facade scale of the case study. Architectural inputs were geometry of the facade, size of the envelope, curvature, active BIPV area and the location of the installed upper-window photovoltaic band. These were based on architectural records, field photos, and Rhino geometric remodeling. The climatic inputs were based on EPW weather data that represented the hot, dust-prone semi-arid climate of Sulaymaniyah and were applied to map the irradiance and evaluate the annual energy. The key environmental inputs were location, direct and diffuse solar radiation and global horizontal radiation with the radiation analysis also considering north orientation, façade/context geometry, analysis grid settings and surface offset parameters. In the PVsyst cross-check, the closest available Meteonorm-based data of Ḩayy Tuwī Ḩamalayk, Iraq, was taken with a fixed vertical facade position. The photovoltaic inputs were defined separately for the as-built and expanded baseline. The installed upper-window system is made up of 900 semi-transparent monocrystalline panels with an active glazing area of about 752.99 m², an installed capacity of 232 kWp DC, and two 110 kW Huawei SUN2000-110KTL-M0 inverters, providing 220 kW AC. This configuration was used as the validation set-up. The model was expanded to a complete-facade analytical base with the active photovoltaic area of 5,814.6 m². To represent the applied upper-window modules, the installed panel sizes of 0.81 × 1.01 m were used, while the reconstruction of the expanded facade was made with facade-aligned units of 3.64 × 1.01 m in order to obtain continuous coverage of the curving envelope. The inputs of the baseline material and system were solar transmittance, visible light transmission, absorptance, thermal conductance and temperature-related photovoltaic parameters, assigned based on available manufacturer information and published values of semi-transparent crystalline BIPV systems. The electrical assumptions were also in accordance with the nature of the existing tower subsystem such as representative DC to AC ratio of 1.05 and inverter performance that was in line with the installed Huawei units. The monocrystalline efficiency was calibrated to be 34% in the modelling input and not a generalized commercial value of semi-transparent facade-integrated BIPV modules. To match the reported annual output, this value was determined by repeated calibration trials to reproduce the annual energy. It should be understood as a case-specific equivalent simulation parameter, and not a typical market efficiency parameter. In the case of the full-facade baseline, 10% soiling coefficient based on literature was selected and 8% and 12% were taken as lower and upper sensitivity limits. The facade in PVsyst was simplified as a fixed vertical plane with no near-shading definition to facilitate the tool-to-tool comparison in simplified exposure conditions. The secondary-layer scenarios were not modelled as complete resolved material systems, but rather they were presented by literature-based comparative energy-gain factors to the validated base. Table 2.

**Table 2:** Main data inputs and modelling parameters used for the Smart Health Tower BIPV analysis.

Input category	Parameter	Value/description	Source/basis	Role in the model
Case study	Building	Smart Health Tower, Sulaymaniyah	Case-study selection	Building used for validation and façade-scale analysis
Façade geometry	Building form	Fully glazed, curved high-rise healthcare façade	Architectural documents, on-site photographs, Rhino reconstruction	Defined the architectural envelope for façade-integrated PV analysis
Façade geometry	Geometric inputs	Façade geometry, envelope dimensions, curvature, active BIPV area, and upper-window PV band location	Architectural documentation and geometric reconstruction	Established the façade-scale analytical model
Applied BIPV system	Installed location	Upper-window bands on the two curved façades	Existing built condition	Real applied system used for validation
Applied BIPV system	Installed panels	900 semi-transparent monocrystalline panels	Building/facility information	Basis of the tool-to-reality validation case
Applied BIPV system	Installed active glazing area	752.9 m²	Building/facility information	Validation area of the applied BIPV band
Applied BIPV system	Installed capacity/inverter setup	232 kWp DC; 2 × 110 kW Huawei SUN2000-110KTL-M0	Building/facility information	Electrical characteristics of the applied system
Applied system geometry	Installed panel dimensions	0.81 × 1.01 m	Existing built condition/geometric reconstruction	Defined the geometry of the applied upper-window BIPV band
Validation reference	Reported annual generation	321,000 kWh/year	Building office records	Tool-to-reality validation reference
Full-façade baseline	Active photovoltaic façade area	5,814.6 m²	Rhino-based façade reconstruction	Building-scale analytical baseline
Full-façade baseline	Façade-analysis segments	2,296 Rhino-based output pieces	Rhino–Grasshopper model	Geometric subdivision for façade-scale radiation and PV analysis
Full-façade baseline	Façade-aligned unit dimensions	3.64 × 1.01 m	Rhino-based façade assignment	Used for continuous coverage across the curved full-façade baseline
Climate input	Weather data source	EPW-based climatic dataset representing Sulaymaniyah; PVsyst cross-check used the nearest Meteonorm 8.1 synthetic dataset for Ḩayy Tuwī Ḩamalayk, Iraq	Ladybug weather input and PVsyst site setup	Climatic basis for irradiance mapping and annual energy evaluation
Climate input	Main climatic variables	Location, direct normal radiation, diffuse horizontal radiation, and global horizontal radiation	EPW weather file/imported climate data	Defined solar resource conditions for façade exposure and energy analysis
Radiation-analysis input	Simulation controls	North orientation, façade/context geometry, analysis grid settings, and surface offset parameters	Rhino–Grasshopper–Ladybug workflow	Controlled façade-scale irradiance mapping
Glazing/PV assembly	Baseline PV window type	Semi-transparent crystalline BIPV glazing	Existing system typology and published references	Defined the baseline BIPV glazing system
Material input	Optical and thermal properties	Solar transmittance, visible light transmission, absorptance, thermal conductance, and temperature-related PV parameters	Available manufacturer information and published values	Defined baseline glazing and PV performance behaviour
Electrical input	DC–AC ratio/inverter basis	1.05; aligned with installed Huawei inverter performance	System-aligned electrical assumption	Defined baseline electrical conversion assumptions
Calibrated input	Monocrystalline efficiency value	34% calibrated modelling input	Model calibration against reported annual output	Case-specific parameter used to reproduce observed generation
Soiling assumption	Baseline and sensitivity bounds	10% baseline; 8% and 12% lower- and upper-bound cases	Literature-informed assumption	Represented baseline dust losses and bounded sensitivity testing
Baseline verification	Rhino–Grasshopper–Ladybug result	2,478,305 kWh/year	Primary simulation workflow	Baseline annual yield used in the comparative framework
Baseline verification	PVsyst cross-check result	2,470,459 kWh/year	PVsyst baseline report	Tool-to-tool verification of annual yield
Scenario method	Secondary-layer representation	Literature-derived comparative energy-gain factors applied to the validated baseline	Comparative framework	Used to assess retained secondary-layer interventions

### Digital modelling and simulation workflow

**Fig. 7:**
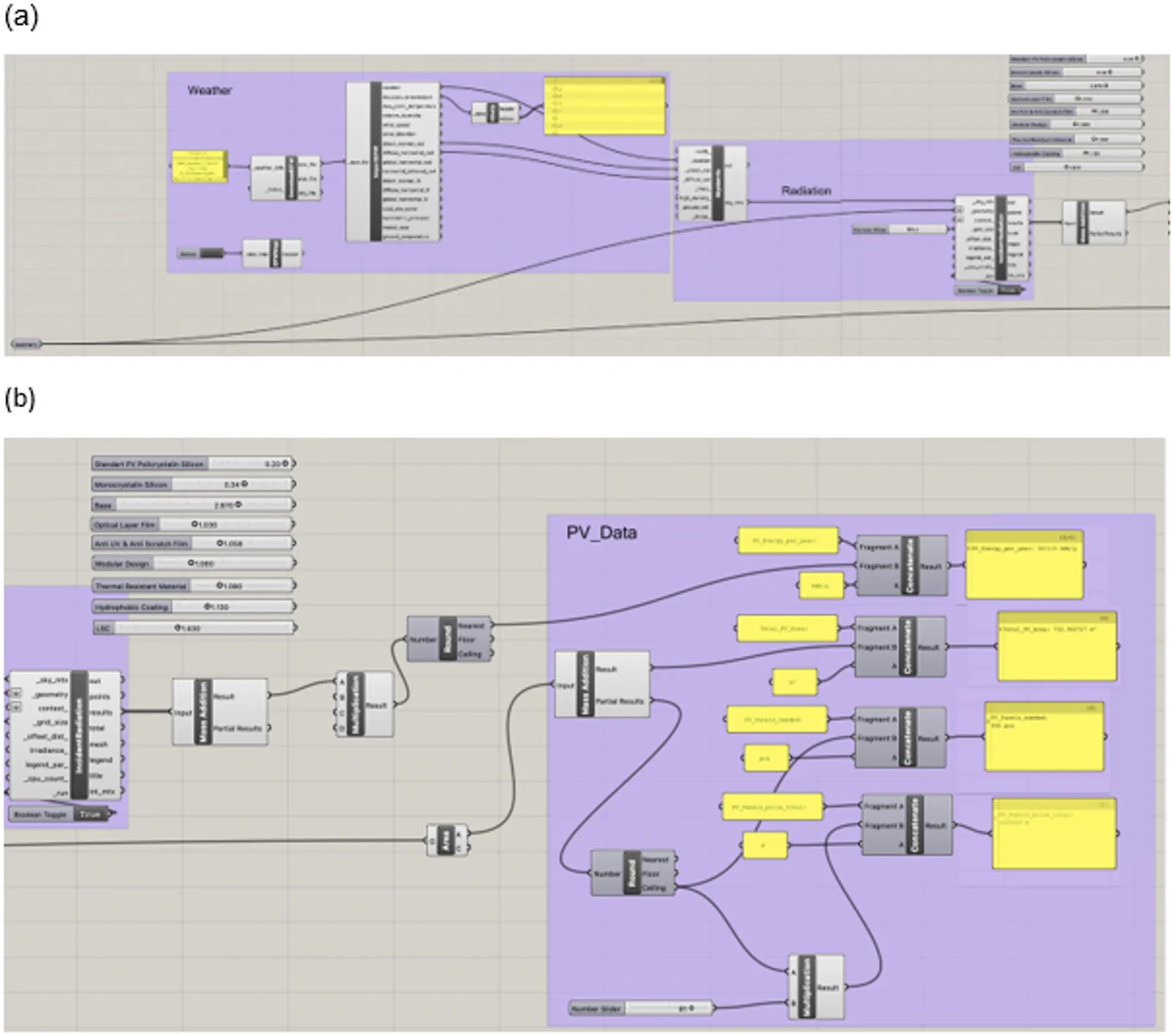
(a & b). Computational workflow to process climatic inputs, simulation of facade solar exposure and the output of annual photovoltaic performances.

The digital workflow combined the geometry of the facade, climatic input, analysis of solar-radiation and photovoltaic output in one single framework of a facade scale, as depicted in Fig. 7 (a & b). The envelope of the Smart Health Tower was rebuilt in Rhino and organized in Grasshopper to parametrize the curved glazed facade and subdivide it into parts depending on the orientation. Climatic information was added using the EPW weather file and computed in Ladybug to create sky matrix and measure the annual and seasonal incident radiation on the non-uniform vertical envelope. It was estimated using the Rhino-Grasshopper-Ladybug workflow on the basis of this radiation layer and compiling the main outputs of annual electricity production, active photovoltaic area and the number of panels. The retained intervention cases or scenarios were compared with the same parametric environment with coefficient-based inputs applied to the validated baseline. A cross-checking of the full-facade baseline in PVsyst was then performed as a tool-to-tool verification step where the façade was characterized as a fixed vertical plane (tilt/azimuth 90°/0°) with Meteonorm 8.1 synthetic weather data at the closest available corresponding to Ḩayy Tuwī Ḩamalayk, Iraq. PVsyst was applied to check the baseline annual energy performance and examined the 8%, 10% and 12% soiling-coefficient scenarios under the same baseline assumptions. In this regard, Rhino-Grasshopper-Ladybug was used as the main facade-based design and simulation environment and PVsyst was used as the secondary check of the entire facade baseline. The model lacked explicit near shading modelling, interlayer airflow modelling, convective heat transfer modelling, internal cavity dust deposition, and layer-by-layer optical losses.

### Tool-to-reality (tool-to-tool) verification and validation of the baseline model

The baseline model were validated in two stages. To compare the as-built upper-window BIPV facade with the reported yearly electricity generation of 321,000 kWh/y, the as-built performance of the system achieved 321,685 kWh/y in Rhino-Grasshopper-Ladybug and 321,115 kWh/y in PVsyst, which is quite similar to the actual performance of the system. Following this tool-to-reality validation, the model was expanded to the full-facade baseline and again cross checked to a second simulation platform. Based on the same baseline assumptions, Rhino based on Grasshopper and Ladybug projected 2,478,305 kWh/y, PVsyst projected 2,470,459 kWh/y giving an absolute difference of 7846 kWh/y and a relative difference of 0.32%. together, these findings suggest that the original model was solid enough to allow the further comparative analysis of the retained secondary-layer situations. Table 3.

**Table 3:** Summary of baseline validation and verification.

Validation stage	Reference/platform	Annual energy output (kWh/year)	Difference from reference (kWh/year)	Difference (%)
Tool-to-reality	Reported upper-window BIPV system	321,000	—	—
Tool-to-reality	Rhino–Grasshopper–Ladybug simulation of upper-window BIPV system	321,685	+ 685	+ 0.21
Tool-to-tool	Rhino–Grasshopper–Ladybug full-façade baseline	2,478,305	—	—
Tool-to-tool	PVsyst full-façade baseline	2,470,459	−7,846	−0.32

**Fig. 8:**
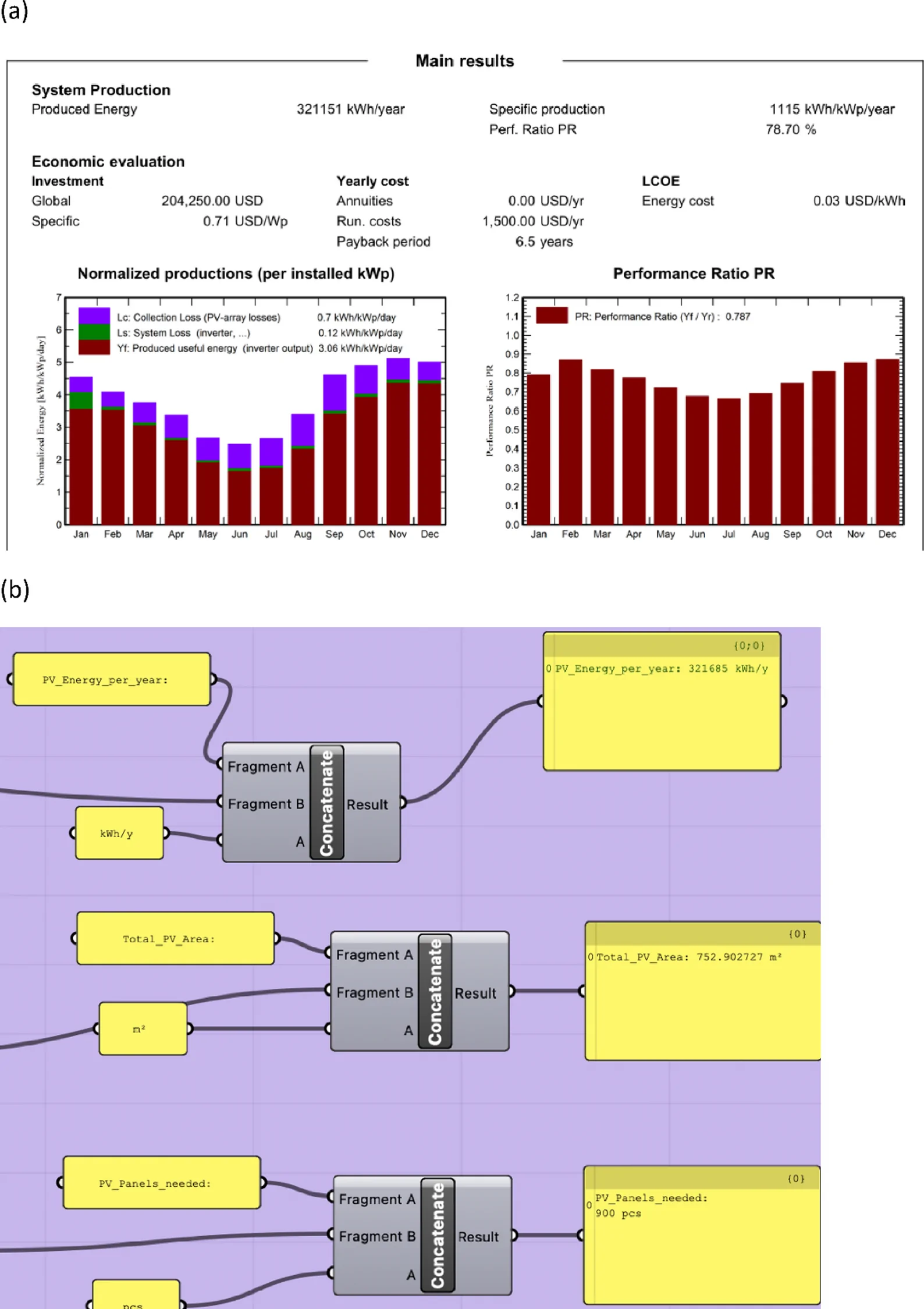
**(a**,** b)** Verification of the simulation framework. **(a)** PVsyst baseline performance is used as tool-to-tool electrical benchmarking. **(b)** Built-system verification of the existing BIPV façade belt integrated in the upper window band of the Smart Health Tower, used as tool-to-reality validation.

### Soiling sensitivity analysis of the full-façade baseline

A limited sensitivity analysis was performed to investigate the impact of soiling on the full-façade baseline. The reference case was simulated using the 10% soiling coefficient operating at, and the lower and upper bound case scenarios were 8% and 12%, respectively. In PVsyst, these cases produced annual outputs of 2,475,096 kWh/year for the 8%, 2,470,459 kWh/year for the baseline, and 2,467,531 kWh/year for the 12%kWh/year. The significance of this test was that soiling is one of the major performance variables of BIPV facades in semi-arid and dusty climatic conditions. In the frequent dust deposition, this 12% would eventually become higher and the losses may be even greater if the facade is not cleaned properly. In this regard, the analysis acted as an uncertainty test of the baseline and enhanced the robustness of the latter comparative evaluation. Table 4.

**Table 4:** Soiling sensitivity analysis of the full-façade baseline.

Soiling coefficient	Annual energy output (kWh/year)
8%	2,475,096
10%	2,470,459
12%	2,467,531

### Secondary-layer strategies for energy performance optimization of BIPV windows

The retained secondary-layer scenarios are different but complementary ways to enhance the implementation of BIPV glazing on the facades in the dust-prone semi-arid environments. In this research, each scenario is described as physically plausible intervention, which is in front of the BIPV glazing within a ventilated multi-layer façade concept. Since the literature lacks uniform facade-specific data on warranty, service life and life-cycle cost on all cases, the durability and cost are addressed qualitatively in terms of robustness, maintenance burden, implementation complexity, and operational relevance.

1. **Optical film layer.** This scenario is the application of a very transparent anti-reflective or light-management film/coating on the outer surface of the secondary glazing layer to minimize the amount of reflection and maximize the amount of useful light passing through to the PV glazing. It can be anti-reflective layers made of silica, textured solar glass or polymer films that are transparent. The primary purpose is optical enhancement though some treatments can also minimize dust-related optical loss. It is not very difficult to install, and its long-term performance is determined by the scratch resistance and abrasion to cleaning resistance, and it is a low- to middle-cost solution whose longevity is determined by the quality of coating.

2. **Anti-UV/anti-scratch layer.** The principle of this scenario involves the application of a transparent protective outer layer to maintain transmittance and surface integrity during time by blocking the UV radiation and mechanical wear. Common substances are hard-coated PMMA or polycarbonate, ETFE front sheets and oxide-coated glass. Its main role is retained surface quality and not immediate energy gain as it assists in minimizing yellowing, embrittlement and abrasion. It is thus a middle priced defensive measure that has a value in long term performance retention.

3. **Modular design logic**. This is mostly an assembly strategy and operational strategy as opposed to a single layer of material as in the other cases. It consists of subdivision of the secondary facade into small replaceable parts which can be washed, repaired or substituted without disrupting the entire facade. Its primary purpose is performance recovery in the form of maintenance, especially in high-rise BIPV facades, where access and maintenance are challenging. It is more demanding in detailing and coordinating, but has higher resilience to long term maintenance.

4. **Thermal-resistant layer**. This is a scenario where an additional layer is added to lower the operating temperature of the PV by means of passive thermal moderation, and is achieved by spectrally selective glazing, low-emissivity coated glass, other thermally protective materials. Its principal operation is minimization of the conversion loss with temperature at high irradiance. Since it has to be incorporated without any effect on transparency, weight and maintainability, it is more complicated than a plain film and should be considered as a moderate- to high-priced intervention, whose sustainability depends on the chosen material system.

5. **Hydrophobic coating layer**. This scenario is made up of a transparent water-repelling coating applied on the outermost layer of the secondary layer to avoid dust adhesion and enhance self-cleaning by rain or rinse water. Common ones are siloxane-based, paraffin-based, fluorinated or nano-engineered, and occasionally have anti-reflective capabilities. Its major action is anti-soiling behavior, which assists in maintaining optical transmittance and loss reduction due to dust between cleaning operations. It is quite simple to implement, yet its functionality is determined by the hardness and resistance to abrasion of the coating and thus periodic renewal may be required.

6. **LSC-based concentrating layer**. This scenario represents the most developed optical concept. A luminescent solar concentrator is a transparent polymer or glass waveguide, doped with fluorescent species, which absorb solar radiation and re-emit it to PV cells or a receiving PV surface. In this study, it is interpreted as a transparent secondary concentrating layer placed in front of the BIPV glazing. The primary process is the concentration of light and spectral control with the advantage that it can utilize direct and diffuse radiation. Even though it has good architectural applicability and good performance capability, its life cycle is questionable due to long-term behavior being related to dye stability, matrix ageing, UV exposure, humidity and thermal cycling.

Altogether, the six scenarios can be discussed as six principal functional routes towards enhancing BIPV facade functionality, which include optical enhancement, retained surface quality, maintenance-related recovery, thermal moderation, anti-soiling behavior, and light concentration. This difference reveals the fact that the cases that are retained are not interchangeable coating, but various architectural and material interventions that have varied logic of installation, durability, and functioning consequences. The section is methodologically defined their physical meaning before they are represented by literature-informed comparative energy gain factors.

**Table 5:** Functional definition and implementation characteristics of the retained secondary-layer scenario. **Source**: Developed by the author from the literature synthesis of the retained secondary-layer scenarios.

Scenario	Typical material/system	Application to secondary layer	Main mechanism	Main façade benefit	Durability/endurance	Cost level	Practical note
Optical film layer	Anti-reflective coated glass or transparent optical film	Applied to the outer face of the secondary glazing	Optical enhancement	Improves light transmission to the BIPV glazing	Moderate; depends on scratch and abrasion resistance	Low–moderate	Suitable where high transparency and minimal visual change are required
Anti-UV/anti-scratch layer	Hard-coated polycarbonate, PMMA, ETFE, or UV-protective coated glass	Used as a protective front layer	Retained surface quality	Preserves transmittance and reduces surface degradation	Moderate–high; depends on UV stability and coating hardness	Moderate	Useful where dust abrasion and repeated cleaning are expected
Modular design logic	Segmented replaceable façade units or demountable secondary-layer panels	Applied at assembly level	Maintenance-related performance recovery	Improves access, replacement, and cleaning operability	High at system level if detailing is robust	Moderate–high	Most relevant for high-rise façades with difficult maintenance access
Thermal-resistant layer	Spectrally selective glass, coated glazing, or passive thermal-regulation layer	Incorporated into the secondary pane/interlayer	Thermal moderation	Reduces temperature-related performance losses	Moderate–high; depends on material type and long-term thermal stability	Moderate–high	Requires careful integration to avoid compromising transparency or weight
Hydrophobic coating layer	Water-repellent nano- or polymer-based coating	Applied to the outermost exposed surface	Anti-soiling behaviour	Reduces dust adhesion and helps preserve transmittance	Moderate; may decline under abrasion and repeated cleaning	Low–moderate	Easy to apply but may require renewal over time
LSC-based concentrating layer	Transparent luminescent waveguide in glass or polymer form	Integrated as a transparent concentrating layer in front of the BIPV glazing	Light concentration and spectral management	Enhances useful light harvesting and supports diffuse-light utilisation	Variable; depends on photostability, UV resistance, and matrix ageing	Moderate–high	Highest innovation potential, but also the greatest material-performance uncertainty

The retained scenarios are not interchangeable surface treatments since they are different strategies of architecture and materials, as illustrated in Table 5. They are used in optical enhancement, anti-soiling action, retained surface quality, maintenance-related recovery, thermal moderation and light concentration. In this respect, the coefficients of the adopted scenarios are indicative of various physical means of improvement rather than a generic coating effect. The strategies in this study were conceptually placed in a ventilated secondary-layer facade system, and airflow, convective heat transfer and dust deposition in the internal cavity was not directly simulated.

### Comparative energy-gain-factor technique in secondary-layer conditions

After the functional definition of the retained secondary-layer interventions, they were evaluated in terms of performance by comparative energy-gain-factor approach in reference to the tested full-façade baseline. According to this approach, every intervention was given a literature-based percentage increase and was applied to the original energy output per year in order to estimate its relative effect at the scale of the facade. The percentage coefficients adopted were 3.0% for the optical film layer, 5.8% for the anti-UV/anti-scratch layer, 6.0% for the modular design logic, 8.0% for the of the thermal-resistant layer, 13.0% for the hydrophobic coating and 43.0% for the LSC-based concentrating layer. These were computed values based on direct performance gains or where comparable façade-scale energy data were unavailable, from proxy-based indicators to retained surface quality, maintenance-related recovery, thermal moderation or optical enhancement. The origin of the coefficients adopted is represented in Table 6. This was required due to the fact that the literature does not always present results in directly comparable terms of annual energy scale of the facade, but rather in similar terms such as less soiling loss, transmittance retention, passive-cooling benefit, performance-ratio improvement or optical concentration. accordingly, the validated baseline and the comparative conditions are to be interpreted differently: the baseline was established by the tool-to-reality validation and tool-to-tool verification, while the secondary-layer cases present literature-based comparative projections, rather than completely resolved simulation of a single material system. Reflection, absorption, scattering, thermal penalties and interlayer airflow were not directly simulated but instead they were indirectly accounted for through the adopted literature-derived coefficients.

**Table 6:** Source-by-source evidence used to benchmark the adopted scenario coefficients.

Scenario	Comparable direct gain range (%)	Supporting non-gain metric(s)	Adopted coefficient (%)	Metric type	Evidence status	Literature basis
Optical film layer	3.0–8.0	—	3.0	Direct power/performance gain	Direct	Hossain et al.^18^,: ~3% performance improvement for combined anti-dust + anti-reflection treatment; Said et al.^38^,: 4–8% power-output increase for textured anti-reflective glass.
Anti-UV/anti-scratch layer	N/A	< 3% transmittance loss after UV testing; about 6% power drop after UV testing; UV-blocking improvement up to 36.0–54.3%	5.8	Protection/retained-performance proxy	Proxy-supported	Zahid^22^, and Johansson et al.^23^, support UV protection and retained performance; the adopted 5.8% is proxied from Al Bakri et al.^55^,(5–6% outdoor power increase for coated panels) and Mehdi et al.,) and^24^(5.8% higher energy yield/PR for a desert-adapted protective module).
Modular O&M strategy	N/A	PR improvement from 91.7 to ≥ 95; annual soiling loss 4.3–7.5%; cleaning benchmark up to 32.27% recovery in a heavily soiled plant	6.0	O&M recovery proxy	Proxy-supported	Walker^15^, supports performance recovery through O&M and soiling-loss avoidance; Hammoud et al.^16^,; provides an upper cleaning-recovery comparator; Bao and Xiang^35^ and Salvalai and Zhao^39^ support the architectural and energy relevance of modular/prefabricated BIPV integration.
Thermal-resistant layer	0.74–11.4	Temperature reduction/passive cooling effect	8.0	Direct power/energy gain	Direct	Hamdan^40^,: 0.74%, 2.14%, and 5.77% power increase for passive-cooling variants; Ramos et al.^41^,;: 7.9–11.4% electricity-generation increase for PCM-assisted passive thermal regulation.
Hydrophobic coating layer	9.6–14.3	47.6% power-loss reduction; 50.2% dust-deposition reduction; 50% transmittance-loss reduction in dry conditions	13.0	Direct gain with additional loss-reduction support	Direct	Chala et al.^21^,: 9.6% improvement over regularly cleaned panels and 13% power improvement over uncoated panels; El-Mahallawi et al.^56^,;: up to 14.3% electrical-efficiency improvement; Tuo et al.^20^,; and de Jesus et al.^19^, strengthen the anti-soiling mechanism basis.
LSC-based concentrating layer	26.0 to > 100	Optical effective concentration 1.23–1.31	43.0	Direct power gain	Direct	Nie^29^, reported a 1.26 power gain over the bare solar cell in a BICPV smart window, equivalent to 26% gain. Zhang^30^, reported a highest power gain of 1.50, equivalent to 50% gain, for a bottom-mounted LSC. Most importantly, Griffini et al.^57^, reported a maximum power conversion efficiency gain of about 43% for optimized perylene-based LSC devices. Therefore, the adopted 43.0% coefficient from the present simulation lies within the direct LSC literature

### Performance indicators and baseline seasonal production profile

The validated full-façade baseline and the retained comparative interventions were measured through four indicators that included annual energy yield, specific yield, performance ratio, and relative energy gain. Annual energy yield is total electricity generation in one-year, specific yield scales the output depending on installed capacity, performance ratio is the overall system performance in the PVsyst-based baseline and soiling-sensitivity evaluation, and relative energy gain expresses the projected enhancement of each intervention on the validated baseline. The results of the PVsyst cross-check of the baseline under the adopted 10% soiling coefficient were 2,470,459 kWh/year of the specific yield, 1,116 kWh/kWp/year of the performance ratio of 78.75%. These were the values that were utilized as the primary baseline of interpretation.

**Fig. 9:**
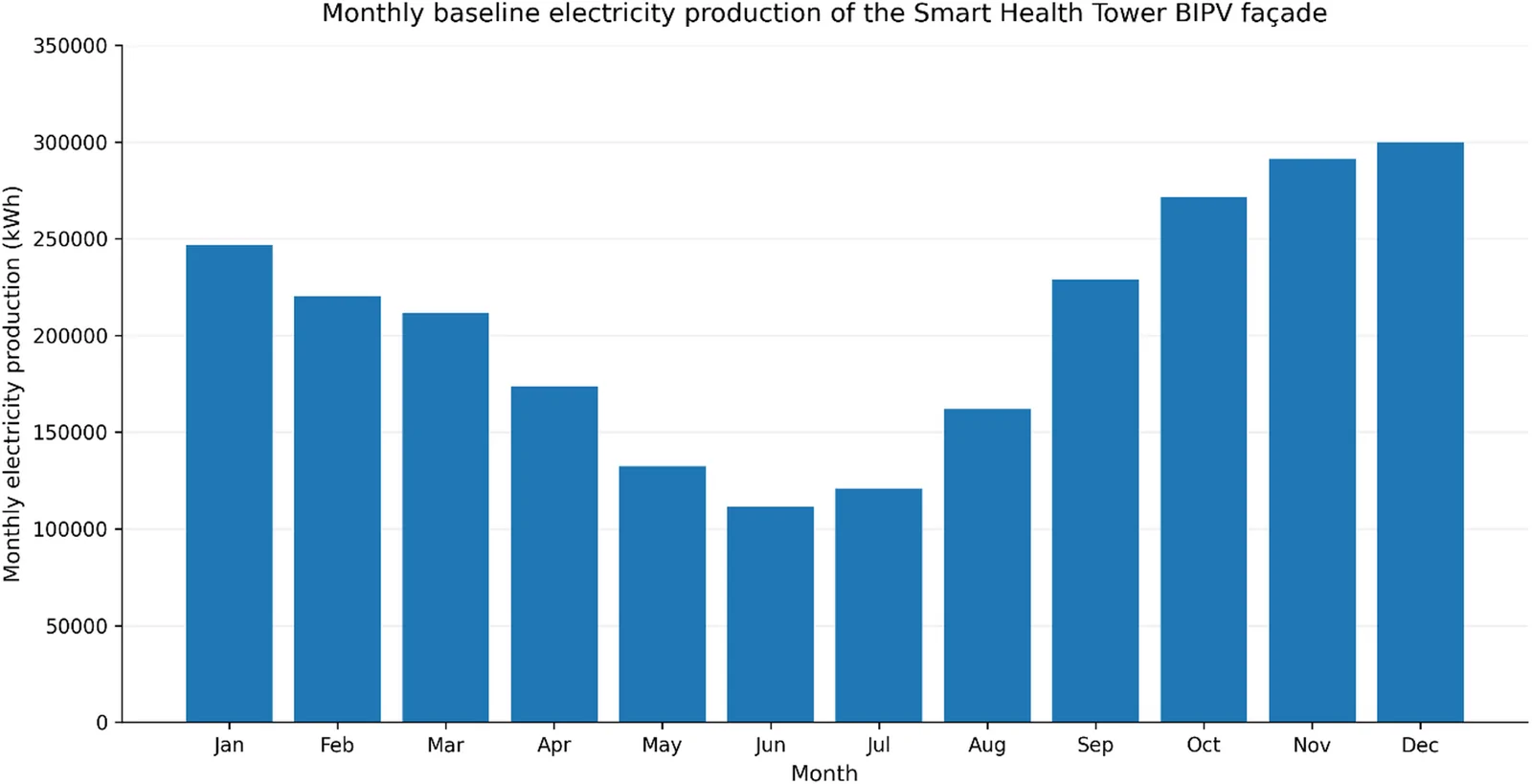
Monthly electricity production of the full-façade baseline BIPV configuration under the adopted 10% soiling condition.

**Fig. 10:**
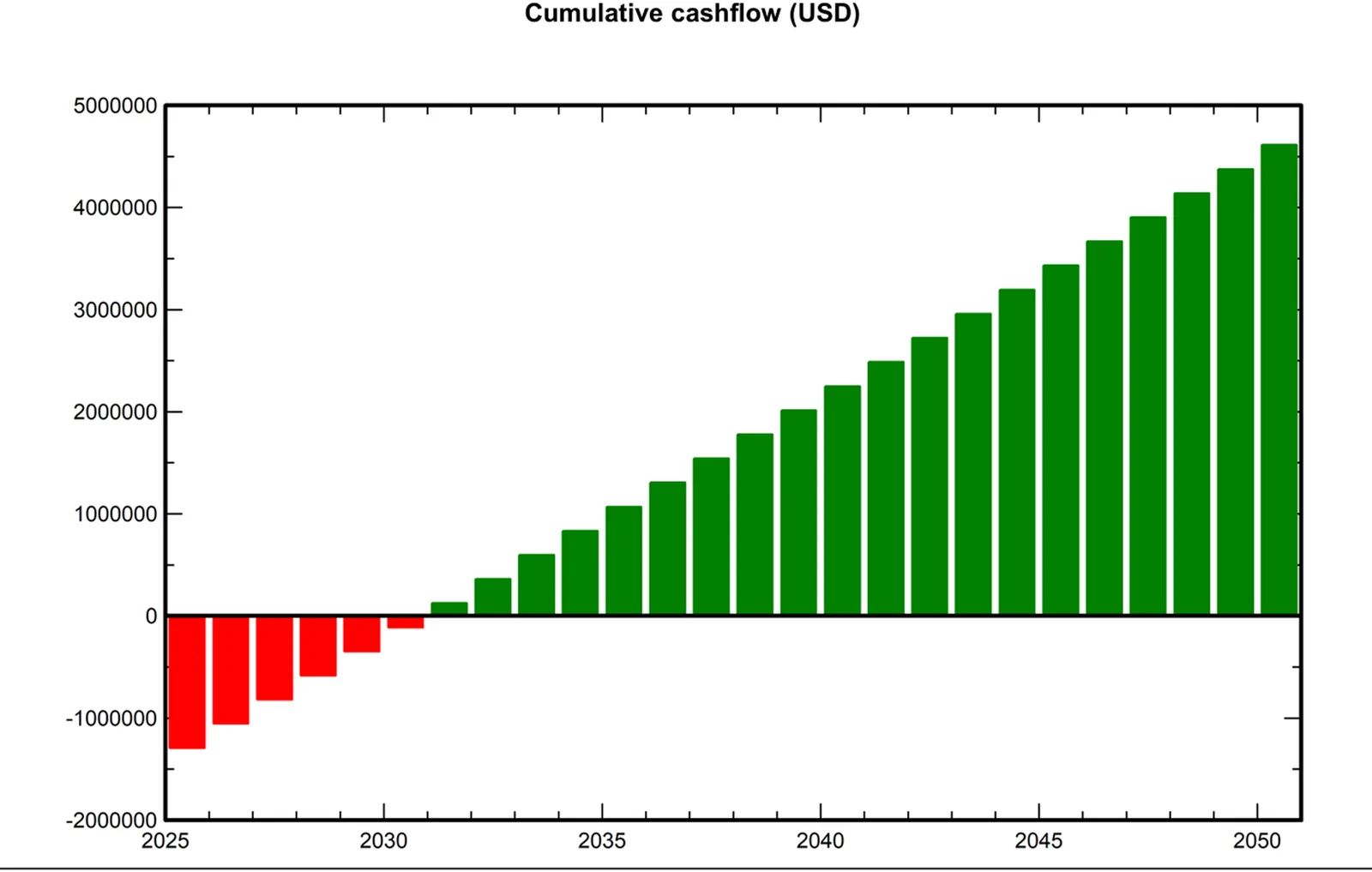
PVsyst-derived cumulative cashflow for the baseline full-façade BIPV configuration under the adopted financial assumptions, indicating a simple payback period of approximately 5.5 years and continued positive return over the 25-year project period.

The monthly electricity generation was used to check the base seasonal profile. As indicated in Figs. 8, 9 and 10 production was lowest in June and July (111,486 and 120,949 kWh) and highest in November and December (291,332 and 299,905 kWh). This implies that the vertical BIPV facade worked better with lower solar angles of cooler seasons as compared to summer. The analysis of seasonality was limited to the baseline case whereas comparison of scenarios was made in terms of annual indicators and gain in energy in comparative terms.

### Preliminary cost analysis

Preliminary cost analysis was carried out only for the validated full-facade baseline. According to the economic output of PVsyst, the system had a total installation cost of USD 1,292,930, annual operating costs of USD 15,800/year, cost of energy USD 0.0273/kWh and a simple payback period of 5.5 years. The analysis also showed net present value of USD 4,611,740.11, internal rate of return of 17.98% and 356.7% return on investment after 25 years. Such findings are valid only to the base scenario and can only be considered as preliminary as the comparative techno-economic evaluation of the scenarios of the retained secondary-layer cases as they outside the scope of the study.

### Uncertainty, assumptions and methodological limitations

This study was carried out as a validated façade-scale framework rather than a complete multi-physics simulation. In spite of the fact the baseline was enhanced with tool-to-reality validation and tool-to-tool validation, the remaining secondary-layer interventions were modelled with literature-based energy-gain factors applied on the validated full-façade baseline. The results can then be taken as a comparative projection instead of a real simulation of the entire optical, thermal, and airflow behavior. The literature-informed soiling coefficients were used as 10% for the baseline and 8% and 12% as the limited sensitivity testing. The suggested secondary-layer cavity had also been conceptualized, without any particular airflow simulation, convectional heat transfer, or internal dust deposition. The proposed secondary-layer cavity was also treated conceptually, without explicit simulation of airflow, convectional heat transfer, or internal dust deposition. Besides, the coefficients adopted were taken out of varying types of literature evidence and thus they must be interpreted as comparative representations rather than universal constants. The economic analysis was limited to the baseline full-facade setting only, and the absolute findings are still specific to the Smart Health Tower. However, the overall framework can be applied to other glazed high-rise structure in dust-prone semi-arid climates.

## Results and discussion

### Annual performance comparative and baseline validation of the BIPV system which is incorporated into the facade

The validated full-facade baseline established the Smart Health Tower envelope as the reference case to perform the comparative evaluation and made sure that the envelope can be used as an energy-generating system on the building scale. In the Rhino -Grasshopper model, the monocrystalline no-layer baseline generated 2,478,305 kWh/year, and PVsyst cross-check generated 2,470,459 kWh/year, also with close agreement in the validation of the installed upper-window BIPV band. On this validated reference, all the scenarios retained in the secondary-layer had enhanced annual electricity generation. Output increased to 2,552,654 kWh/year for the optical film layer, 2,622,047 kWh/year for the anti-UV/anti-scratch layer, 2,627,004 kWh/year for the modular design logic, 2,676,570 kWh/year for the thermal-resistant layer, 2,800,485 kWh/year for the hydrophobic coating, and 3,543,976 kWh/year for the LSC-based concentrating layer. These results correspond to gains of 3.0%, 5.8%, 6.0%, 8.0%, 13.0%, and 43.0%, respectively, relative to the validated monocrystalline baseline. These results of the scenarios indicate that the energy performance of BIPV glazing is not fixed at the installation point but can be substantially enhanced using the secondary-layer design. Small-scale interventions yielded significant benefits, whereas measures that were associated with dust mitigation, thermal response, and light management brought greater benefits. This architecturally significant, as it demonstrates that the architectural choices of the facade at the stage of the layer structure, materials and surface treatments, and optical regulation can enhance the contribution of the operational energy of high-rise BIPV envelopes directly. Table 7.

**Table 7:** Comparative annual performance of the retained scenarios relative to the monocrystalline baseline.

Scenario	Annual energy (kWh/y)	Specific yield (kWh/m²·y)	Gain vs. monocrystalline baseline (%)
Monocrystalline baseline	2,478,305	426.2	0.0
Monocrystalline + Optical film layer	2,552,654	439.0	3.0
Monocrystalline + Anti-UV/anti-scratch layer	2,622,047	450.9	5.8
Monocrystalline + Modular design logic	2,627,004	451.8	6.0
Monocrystalline + Thermal-resistant layer	2,676,570	460.3	8.0
Monocrystalline + Hydrophobic coating layer	2,800,485	481.6	13.0
Monocrystalline + LSC-based concentrating layer	3,543,976	609.5	43.0

### PV window integration offsetting significance of energy

The practical importance of PV window integration can be evident when the output of facades is correlated to the real demand in hospitals. In July, when the demand of the hospital electricity was about 759,000 kWh, the baseline reduced the load by approximately 16.0%. In the retained secondary-layer cases this estimated offset increased to 16.5% of optical film layer, 16.9% for the anti-UV/anti-scratch and modular cases, 17.3% for the thermal-resistant case, 18.1% for the hydrophobic coating, and LSC case had an estimated offset 22.9% respectively. These values have been calculated proportionately on the annual comparative gains. These findings demonstrate that in high-rise health facilities, where the roof area alone is not large enough to make any significant contribution in terms of solar power, the facade may be a useful energy-producing surface. They also outline that the contribution of secondary-layer optimization can additionally make BIPV windows more advantageous with respect to operational energy in dusty semi-arid conditions.

## Conclusion

This study demonstrated that the energy efficiency of BIPV windows in dust-prone semi-arid environments can be enhanced by means of optimization of the facade, rather than photovoltaic integration alone. Based on the Smart Health Tower case study, the validated full-facade baseline generated approximately 2.48 GWh/year, which proves that BIPV glazing will be able to contribute significantly to the electricity supply of high-rise buildings. The close agreement between the simulated and reference outputs were also justified the validity of the adopted modelling framework. The findings also indicated that the secondary-layer design has the potential to enhance the performance of the facade. Among the retained scenarios, the hydrophobic coating proved to be one of the strongest practically transferable options and the LSC-based case yielded the highest projected gain. Operational wise, the verified baseline reduced the July electricity demand of the hospital by approximately 16.0% and this increased to 22.9% under the LSC scenario. In conclusion, the study proves that the concept of PV window integration can be perceived as an energy- offsetting facade concept instead of a symbolic sustainability feature. In high-rise buildings, where roof area is limited, the vertical façade can serve as a productive energy surface, and its functionality can be enhanced by climate-responsive second-layer design.

## Data Availability

Data availability: The findings of this study are supported by data extracted from published literature and the simulation results generated by the authors using Rhino, Grasshopper, Ladybug, and PV-syst workflows, and climate input data were obtained from publicly available sources (EPW weather files) and associated performance tools. The corresponding author will provide derived datasets and key simulation outputs upon reasonable request.
